# Appendiceal Abscess Within an Amyand’s Hernia: A Unique Case Encountered During Laparoscopic Appendicectomy

**DOI:** 10.7759/cureus.90710

**Published:** 2025-08-22

**Authors:** Hesham Aowad, Brian Sheridan, Gisella Salerno

**Affiliations:** 1 Surgery, Wexham Park Hospital, Frimley Health NHS Foundation Trust, Slough, GBR; 2 Surgery, American University of the Caribbean School of Medicine, Cupecoy, SXM; 3 Colorectal Surgery, Wexham Park Hospital, Frimley Health NHS Foundation Trust, Slough, GBR

**Keywords:** amyand’s hernia, atypical appendicitis, congenital inguinal hernia - amyand's hernia- caecum- appendix, rare complication of appendicitis, recurrent inguinal hernia

## Abstract

The presence of the appendix within a hernia sac is known as Amyand’s hernia. Amyand’s hernia is frequently diagnosed intraoperatively, as preoperative diagnosis is exceedingly difficult. Concomitant acute appendicitis is a rare complication of Amyand’s hernia. We present a case of an 18-year-old male who was admitted to the emergency department with acute appendicitis. A laparoscopic appendicectomy was performed, during which it was noted that a portion of the appendix had herniated through the internal inguinal ring. The appendix was removed from the inguinal ring. An abscess cavity was washed and shown to have no further contents. An appendicectomy was completed without repairing the inguinal hernia. This case represents a rare instance of an Amyand's hernia in which acute appendicitis was the preoperative diagnosis.

## Introduction

The presence of the appendix within a hernia is known as Amyand’s hernia, and accounts for less than 2% of inguinal hernia cases [1]. The occurrence of acute appendicitis within an Amyand’s hernia is exceptionally rare, representing approximately 0.1% of all cases of acute appendicitis [2]. Intraoperative identification of a vermiform appendix in the inguinal ring is the leading method of diagnosis. A recent literature review cited that only 23.1% of cases were diagnosed preoperatively, all of which were diagnosed with the help of imaging, mostly using computer tomography [3,4]. In both acute and elective cases, hernia is the primary preoperative diagnosis based on examination findings. The appendix being present within the hernia is an incidental finding. A minority of Amyand’s hernia cases have a preoperative diagnosis of appendicitis, representing only 11.7% of reported cases. Manatakis et al. reviewed 442 cases and reported an age range between four days and 92 years with a mean age of 34.3 years ± 31.9 years; 90% of patients were male [3].

Losanoff and Basson [5] described four types of Amyand’s hernia that can be classified through intraoperative identification of appendix features. Type I is described as a normal appearing appendix within the hernia, type II is described as acute appendicitis within the hernia, type III is described as acute appendicitis within the hernia and peritonitis, and type IV is described as acute appendicitis within the hernia with other abdominal pathology outside of the hernia sac [5]. There is controversy about whether the appendix should be removed in the absence of appendicitis, as seen in type I hernias. Advocates of concurrent appendicectomy cite the risk of recurrence of the hernia and the possibility of incarceration requiring a second surgery. In contrast, others state that reduction of the appendix and hernioplasty is sufficient management, requiring no need for removal of the appendix. In the presence of appendicitis with types II-IV hernias, appendicectomy is required, and the conversation focus is on the approach to hernioplasty. The recommendation based on Losanoff and Basson’s classifications is not to use mesh in cases where there is concomitant appendicitis. This is due to the risk of infection of the mesh leading to surgical site infection and the potential of fistula formation.

## Case presentation

We present a case of a patient with a preoperative diagnosis of appendicitis who was found to have a type II Amyand’s hernia intraoperatively. Appendicectomy was performed without hernioplasty. Outpatient follow-up revealed a small, uncomplicated right inguinal hernia, and the patient is being followed as per his request. 

**Figure 1:**
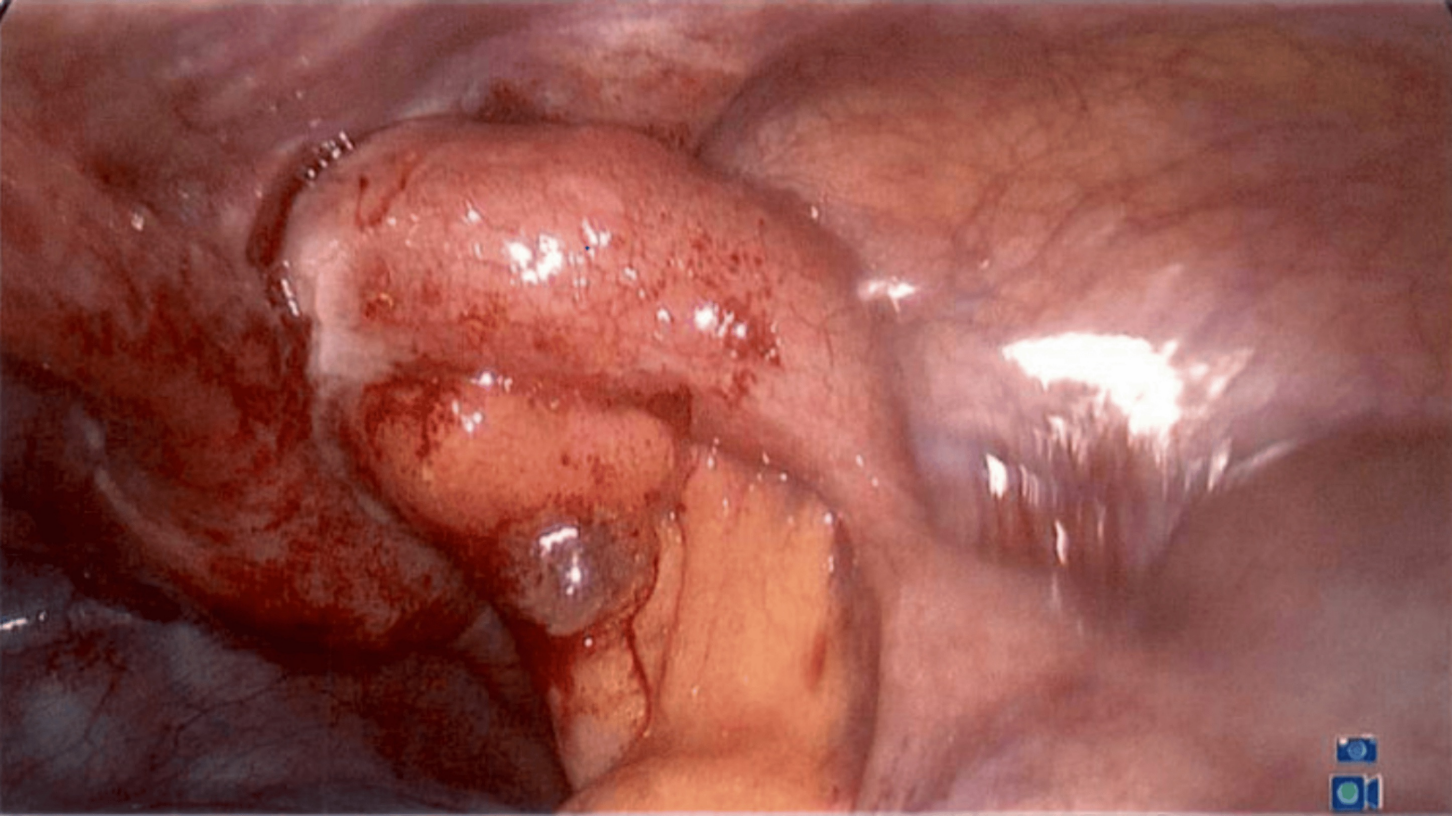
Appendix visualized within the hernia

**Figure 2:**
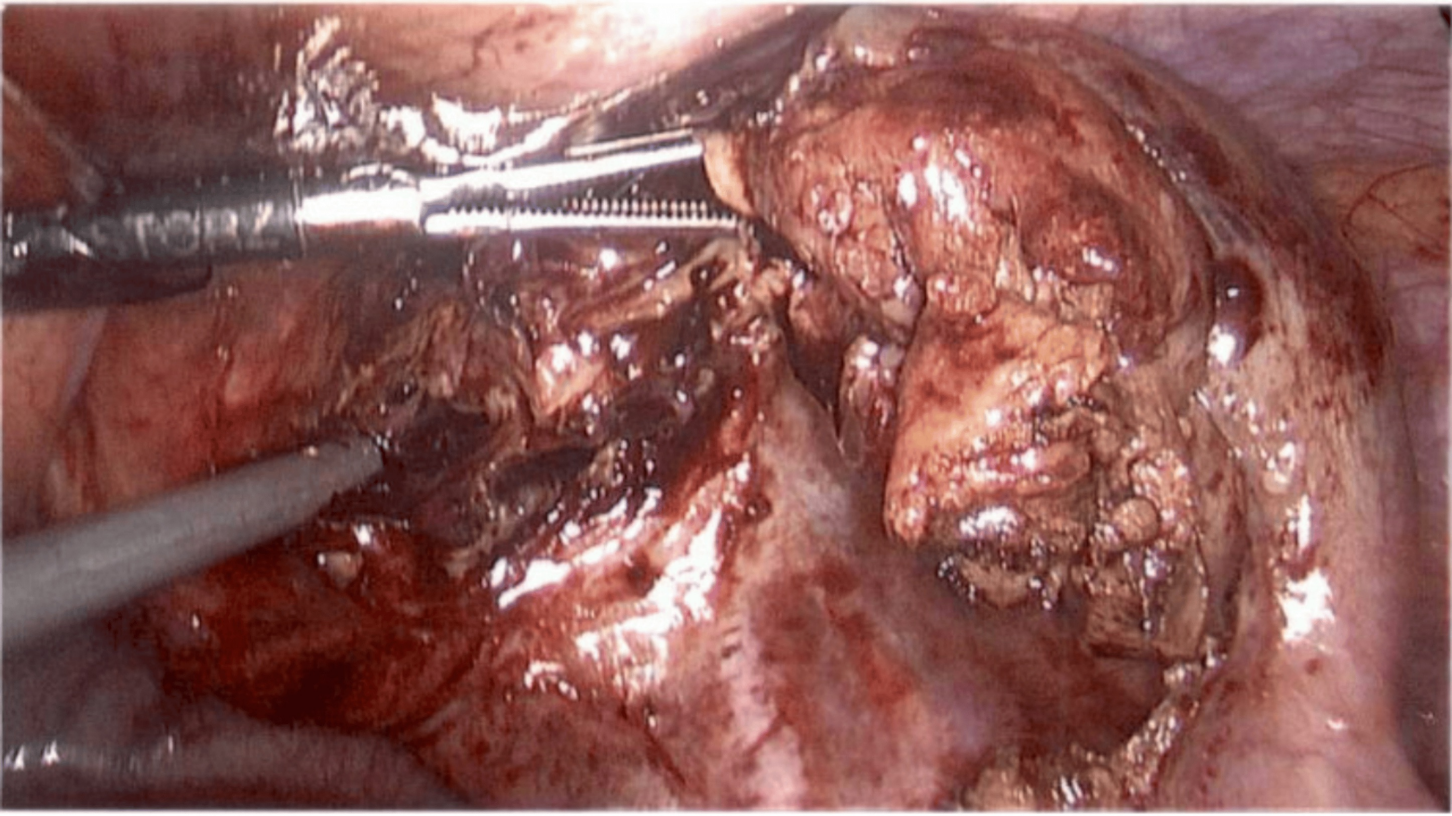
Appendix dissected and removed from hernia

**Figure 3:**
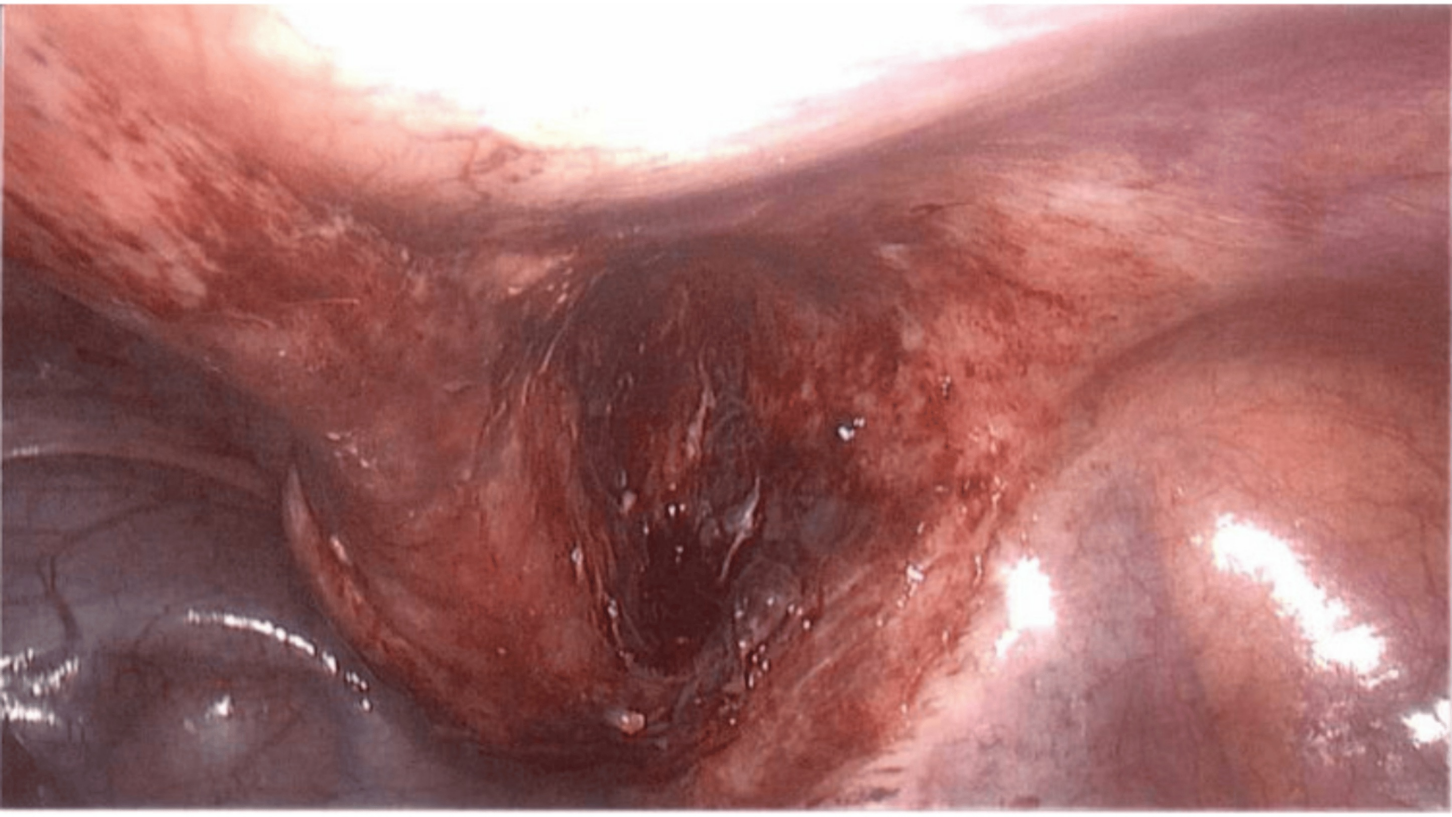
Hernia sac cleaned and shown to be empty

An 18-year-old male with a medical background of type 1 diabetes mellitus presented to the Emergency Department with a one-week history of gradually increasing umbilical abdominal pain that localized to the right iliac fossa. He had fever and diarrhea initially, followed by constipation and dysuria for two days prior to presentation. Physical exam revealed tenderness and guarding in the right iliac fossa, and the diagnosis of appendicitis was made clinically. Preoperative ultrasound confirmed a 22-mm-thickened appendix with a fluid collection distally. The patient consented to a laparoscopic appendicectomy. On laparoscopy, the appendix was seen to be thick and edematous. The tip and shaft appeared to be within the internal inguinal ring, constituting an indirect hernia (Figure 1). Observing the inflamed appendix present within the inguinal ring led to the intraoperative diagnosis of a type II Amyand’s hernia. The mesoappendix was secured and divided, allowing for dissection, freeing the tip and shaft from the inguinal ring. A mild amount of pus came out of the inguinal canal. The internal inguinal ring was visualized (Figure 2). An uneventful appendicectomy and washout of the abscess cavity and pelvis were performed. Visualization of the internal inguinal ring revealed no remaining bowel (Figure 3). Mesh repair of the inguinal ring was not performed at this time. 

Postoperatively, the patient briefly experienced urinary retention, which resolved over the weekend. He was kept over the weekend due to an elevated C-reactive protein at 328 and a white blood cell count of 11,500. He was placed on IV metronidazole and amoxicillin as per trust guidelines to treat intra-abdominal infection. Both urine and blood cultures were obtained, which were negative. Over the weekend, his C-reactive protein and white blood count decreased, and he was discharged on postoperative day 5 on a course of oral antibiotics. 

Follow-up in the clinic, the patient had fully recovered from surgery with no complications and no clinical hernia at that time. However, seven months later, he developed groin discomfort, which was on examination a non-complicated small right inguinal hernia. Notably, the patient mentioned a family history - his father reportedly had a “ruptured appendix in a hernia.” Since the patient was mainly asymptomatic, he preferred conservative management.

## Discussion

The majority of Amyand’s hernia cases present with either a reducible or irreducible hernia as the indication for investigation and surgery. Appendicitis without evidence of hernia only occurs in acute cases and accounts for just 11.7% of preoperative diagnoses [3]. This case is a rare presentation of Amyand’s hernia, where the initial diagnosis was acute appendicitis without any external signs of an inguinal hernia. Following the Losanoff and Basson Classification, we observed a type II Amyand’s hernia, and we performed an appendicectomy without mesh repair of the hernia owing to the risk of infection of the mesh and the grossly inflamed area. The use of a mesh repair hernioplasty is not recommended due to an increased risk of surgical site infection and a possible complication of enterocutaneous fistula [5-7]. While it has been suggested that newly developed prosthetic materials, such as biological mesh, could reduce the risk of infection and allow for hernioplasty in the presence of appendicitis, there is no clinical evidence of this, and research would be needed to support this departure from the current surgical management [6,7].

## Conclusions

This case represents a rare instance of an Amyand’s hernia with concomitant acute appendicitis. The preoperative diagnosis was acute appendicitis, with the hernia being identified intraoperatively. After removal of the appendix and a thorough cleaning of the hernia sac, it was determined that the hernia would not be repaired at this time, in keeping with recommendations of managing an Amyand’s hernia with concomitant acute appendicitis. We tracked the patient’s recovery and followed his care with outpatient visits, which demonstrated that he had recovered well from his operation. We have shown that the management of this case has had no postoperative complications, and the residual hernia can be repaired later without adverse effects.
